# Pediatric urgent care education: a survey-based needs assessment

**DOI:** 10.1186/s12913-019-4241-8

**Published:** 2019-06-14

**Authors:** Xian Zhao, Ioannis Koutroulis, Joanna Cohen, Deena Berkowitz

**Affiliations:** Division of Emergency Medicine, Children’s National Health System/George Washington University, 111 Michigan Avenue NW, Washington, DC 20010 USA

**Keywords:** Urgent care, Residency, Training, Survey

## Abstract

**Background:**

There is an increasing number of pediatric urgent care centers that are largely staffed by pediatric residency graduates. It is unclear if pediatric residency adequately prepares a physician to fully and successfully provide care in an urgent care setting. The goal of this study is to conduct an assessment of urgent care directors’ perceptions of recent pediatric residency graduates’ preparedness to successfully provide pediatric urgent care after graduation.

**Methods:**

This is a 2018 cross-sectional survey of all pediatric emergency medicine division chiefs in the United States and all pediatric urgent care directors who are members of the Society for Pediatric Urgent Care. An electronic survey was distributed consisting of eight multiple choice questions regarding perceived preparedness and knowledge gaps of recent pediatric residency graduates for independent practice in urgent care. Descriptive statistics were used to analyze results and qualitative data were analyzed via an inductive thematic approach.

**Results:**

Forty-two percent (65/154) of surveys were completed. No respondents believed that a recent pediatric residency graduate would be adequately prepared to independently practice in a pediatric urgent care and 81% of respondents recommended some additional training. Most respondents described this training as important (46%) or very important (35%). Most respondents recommended between 6 months and 1 year as the appropriate amount of time to achieve competency.

**Conclusions:**

Despite the growing number pediatric residency graduates staffing pediatric urgent care centers, the majority of surveyed pediatric emergency medicine division chiefs and pediatric urgent care directors do not think that pediatric residency adequately prepares graduates to successfully provide urgent care to pediatric patients. We recommend further exploration of gaps in knowledge of recent pediatric residency graduates as a next step towards developing systems for further training for pediatric residency graduates to gain competency in urgent care management.

**Electronic supplementary material:**

The online version of this article (10.1186/s12913-019-4241-8) contains supplementary material, which is available to authorized users.

## Background

In June 2017, there were 7639 urgent care centers (UCCs) in the United States, an increase in 5% from 2016 [1]. Twenty-one percent of these UCCs are dedicated to the treatment of pediatric patients [2]. There is significant variability in the level of acuity managed at different pediatric UCCs and little is known about the scope, quality and outcomes of care received in pediatric UCCs [3, 4].

The American Academy of Pediatrics Committee on Pediatric Emergency Medicine recommends that urgent care facilities have “experienced staff trained to provide critical support for ill and injured children until transferred for definitive care.” [3] Additionally, they call for educational opportunities directed at pediatric urgent care providers [2]. Many pediatric UCCs are staffed by general pediatricians who were trained in a pediatric residency program [2]. Our primary objective was to qualitatively assess the perceived readiness of pediatric residency graduates to function as independent practitioners in the pediatric urgent care setting. Our secondary objective was to determine the general categories of knowledge gaps deemed most important to achieve competency and to estimate the duration of additional training needed. We conducted a cross-sectional survey of leaders in pediatric urgent care and pediatric emergency medicine, who direct UCCs, to assess perceptions of preparedness, categories of knowledge gaps, and estimated time of additional training needed with regards to recent pediatric residency graduates working in UCCs. Knowledge gap categories were broadly defined as procedural skills, such as suturing of wounds and splinting of orthopedic injuries, and clinical competency, which includes the ability to appropriately diagnose and treat common urgent medical conditions.

## Methods

The survey was created with guidance from an educational researcher using survey development methodology [5]. The final survey consisted of eight questions on the following topics: site demographics, hiring practices, time to competency and need for additional training. Respondents were also asked to rank five urgent care skills in order of importance on a four-point Likert scale. In addition, there were two open-ended questions giving the opportunity for free text responses. The complete survey is included as Additional file 1.

In order to reach the maximum number of urgent care directors, we distributed the survey via email to members of the association of Pediatric Emergency Medicine North American Chiefs (PEMNAC), and to members of the Society for Pediatric Urgent Care (SPUC), an organization formed in 2014 for pediatric urgent care medical directors to develop best practices for ensuring clinical excellence and overall quality of care. A survey reminder was sent 2 weeks after the initial electronic mailing. Respondents were not incentivized for their involvement.

We analyzed the quantitative data using descriptive statistics for central tendency (mean and SD), overall counts and percentages, and frequency distributions. Contingency tables (chi-squared) testing was used to evaluate differences in demographic characteristics between respondents as well as differences in perceived preparedness for independent practice. Analyses were conducted using Excel software (Microsoft, Redmond WA). Qualitative data were analyzed via an inductive thematic approach. This quality improvement study was acknowledged and determined to be exempt from a standard review by the Children’s National Health System Institutional Review Board per our institution’s policy and with the need for consent waived.

## Results

Of the 154 surveys sent, 80 responses were recorded. Fifteen respondents started but did not complete the survey, yielding a completed response rate of 42%. An additional two respondents stated that they were not affiliated with an urgent care center (UCC), leaving 63 total surveys in the final analysis.

### Demographics

The majority of respondents directed urgent care sites that were affiliated with an academic institution (89%). Fifty-four percent surveyed identified their affiliated UCC as within a hospital setting and 35% were freestanding. The remaining 11% were described as freestanding UCC’s, without an academic affiliation. The UCCs surveyed employed physicians with a variety of training, including pediatric residency graduates (89%), pediatric emergency medicine fellowship graduates (56%), emergency medicine residency graduates (30%), and family medicine residency graduates (12%). Forty-seven of our 63 respondents (75%) stated that their site either advertised for or hired a general pediatrician in the past year. Further demographic data are presented in Table 1.

**Table 1:** Demographics of Urgent Care Center Survey Respondents

Characteristic	N (%)
Site	
Academic affiliation, free-standing	22 (35%)
Academic affiliation, within main hospital	34 (54%)
Non-academic, free-standing	7 (11%)
Total	63 (100%)
Location	
Urban	42 (67%)
Suburban	16 (25%)
Rural	0
Mix of urban and suburban sites	5 (8%)
Total	63 (100%)
Physician training^a^	
Pediatric residency	56 (89%)
Pediatric Emergency Medicine fellowship	35 (56%)
Emergency Medicine residency	19 (30%)
Family Medicine residency	8 (12%)
Recruited pediatricians within the past year	
Yes	47 (75%)
No	16 (25%)
Total	63 (100%)
Difficulty recruiting pediatricians	
Very difficult	4 (7%)
Somewhat difficult	33 (60%)
Somewhat easy	12 (22%)
Very easy	6 (11%)
Total	55 (87%)

^a^more than one type per site

### Comfort level with clinical or procedural cases

**Fig. 1:**
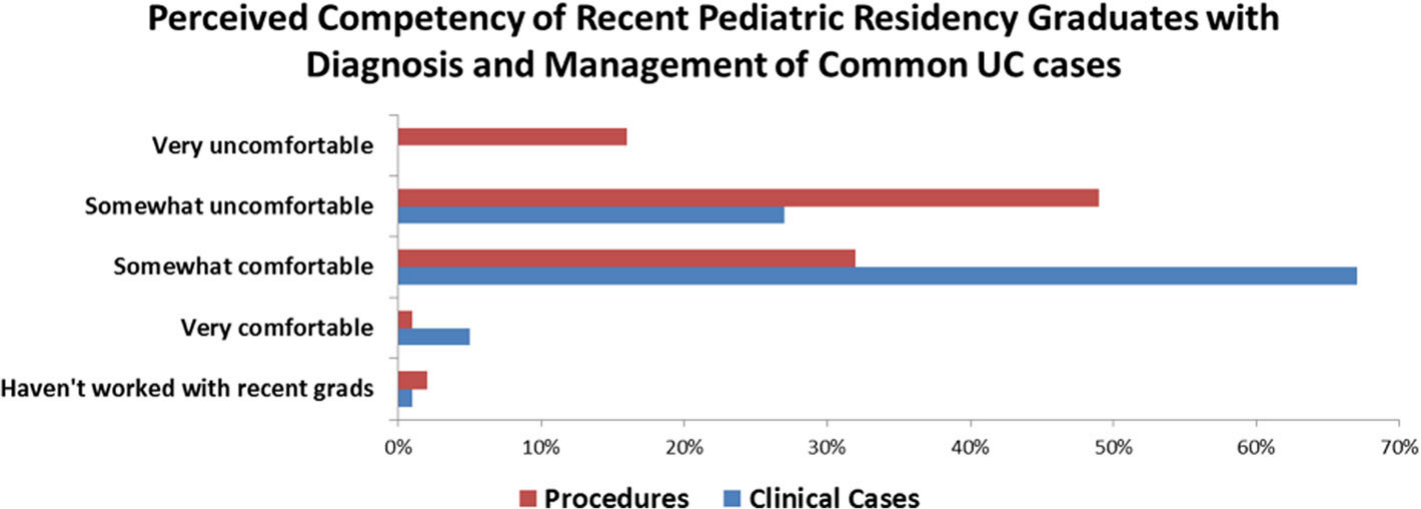
Perceived competency of recent pediatric residency graduates with diagnosis and management of common urgent care cases

The majority of respondents described recent pediatric residency graduates as somewhat comfortable with the diagnosis and management of common urgent care clinical cases (68%), but very uncomfortable (16%) or somewhat uncomfortable (49%) with urgent care procedures. See Fig. 1.

### Time to competence

No respondents believed that a physician would be prepared to work independently in an UCC immediately upon graduating pediatric residency. The respondents recommended additional training ranging from 1 month to 3 years. Fifty-six percent of the survey participants felt that pediatric residency graduates needed more than 6 months of additional training in order to become competent in the field of pediatric urgent care. The average recommended time for additional training was 11 months, and the median and mode of the responses were 12 months.

### Additional training

**Fig. 2:**
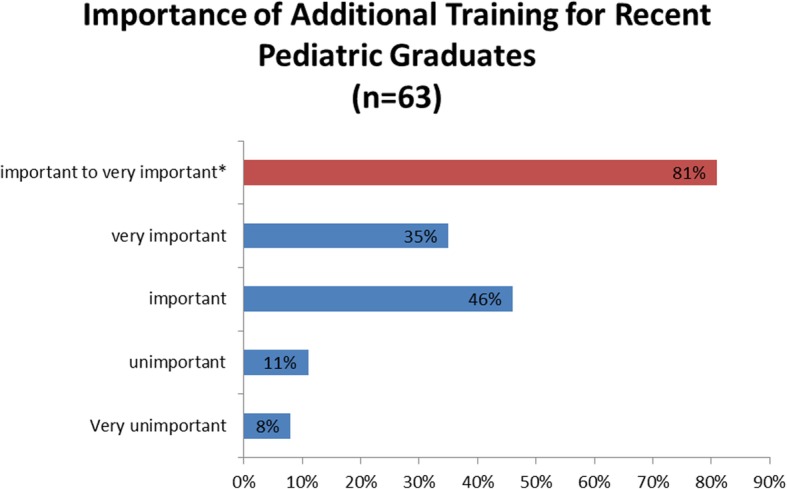
Importance of additional training for recent pediatric residency graduates

Consistent with their assessment that pediatric residency graduates needed additional time to attain competence in urgent care medicine, 81% of respondents considered additional training after residency either important (46%) or very important (35%) when hiring physicians to work in an UCC. Only 19% described additional training as unimportant (11%) or very unimportant (8%). See Fig. 2.

### Necessary skills

**Fig. 3:**
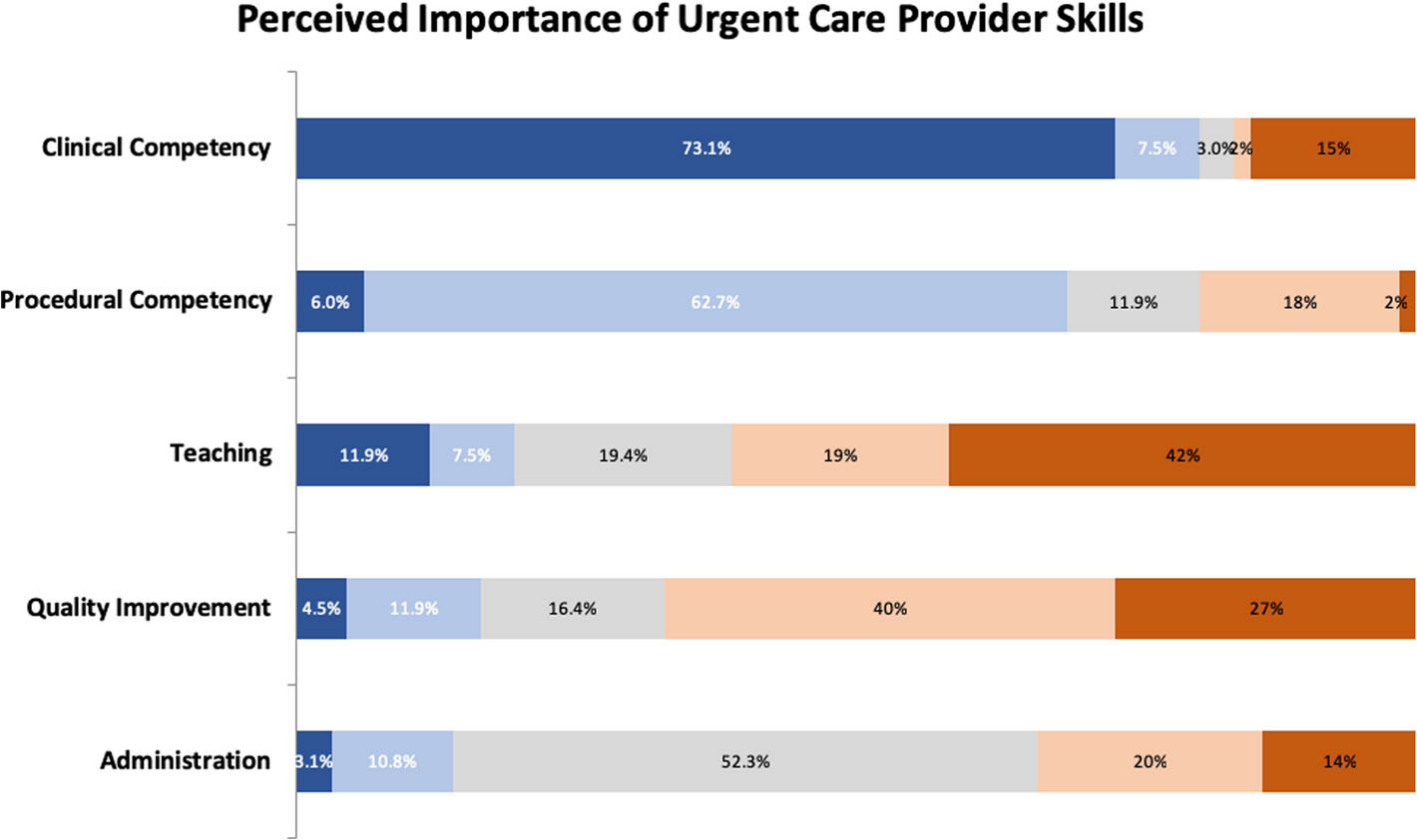
Perceived importance of urgent care provider skills

Most respondents described clinical competency as the most important skill for urgent care providers with procedural competency as the next most important skill. Teaching, quality improvement and administrative skills were perceived as less important. See Fig. 3.

## Discussion

In this needs assessment survey, we found that leaders in the field of pediatric urgent care believe that general pediatric residency graduates need additional training before they can achieve competence in urgent care. The estimated time range for this training varied widely; however, most survey respondents recommended that recent pediatric residency graduates have an additional 6 months to 1 year of training.

The vast majority of survey respondents consider clinical competency the most important skill for a pediatric urgent care provider, followed closely by procedural competency. We believe these aspects of urgent care are best learned in a supervised environment. On the other hand, study participants considered the skills of administration, quality improvement, and teaching as less important. These can be safely acquired on-the-job and, while important, do not directly impact patient care.

Our findings are timely because of the growing demand for urgent care services, due in part to the rising cost of emergency department visits and the shortage of primary care availability [2, 3]. With the expansion of urgent care centers throughout the country, there has also been a concurrent increase in the number of urgent care centers specifically geared toward the care of the pediatric patient. Pediatric residency graduates are the largest portion of the pediatric urgent care center workforce [4].

To our knowledge, this is the first study evaluating the preparedness of pediatric residency graduates to provide care in a pediatric UCC. This adds to the developing set of literature around providing quality, patient-centered care in UCCs. Our results supplement the findings of another study, which examined the effects of establishing an urgent care curriculum and lecture series for residents in the primary care setting. Overall response to the content was positive, but more importantly, referral percentage to subspecialists decreased from 34% before the intervention to 31% after the intervention [6]. Interestingly, our study, which focused on leaders in the field of urgent care medicine, contradicts the findings of another study, which focused on pediatricians’ self-perception. A survey of residency graduates found that, despite limited access to clinical time in the emergency department, 98% of trainees felt that they were well-prepared to manage pediatric emergencies [7]. The gap between the self-perception of residency graduates’ competence and the perception of emergency medicine and urgent care medicine leadership is an interesting finding in and of itself and warrants further investigation.

Pediatric urgent care centers see a wide range of patient acuity and often provide care that encompasses some services normally administered in the primary care office and some traditionally provided in the emergency department. Pediatric urgent care providers are often expected to be competent in fracture management, wound care, abscess drainage and IV placement. In addition, between 1 and 5% of patients who initially present to an UCC will subsequently require transport to an emergency department [4]; consequently, an urgent care provider must be competent to provide stabilization of critically ill children prior to the availability of transport to a higher acuity setting. The ACGME requires only 2 months of pediatric emergency training in 3 years of pediatric residency [8]. Therefore, pediatric residency graduates are often underprepared for this work. Consistent with this, none of the survey respondents feel that a recent graduate of a pediatric residency is ready to provide competent care in an UCC without further training.

The majority of survey respondents recommend that recent pediatric residency graduates have an additional 6 months to 1 year of training to competently provide urgent care. The vast majority of survey respondents consider clinical competency the most important skill for a pediatric urgent care provider, followed closely by procedural competency. The majority of survey respondents consider administrative skills, quality improvement skills and teaching skills as less important. Recently, a new crop of pediatric urgent care fellowships has arisen to meet this demand. Further educational needs assessments will help refine these fellowships to more specifically meet the needs of recent pediatric residency graduates planning on working in pediatric UCCs.

These new urgent care fellowships provide a supervised environment for pediatric residency graduates to gain the clinical and procedural competency needed to work in an urgent care setting. In addition, an academic setting can help fellows gain administrative, quality improvement and teaching skills.

### Limitations

First and foremost, our survey completion rate was only 42%; however, this is comparable to the generally accepted 50% response rate in social research surveys and higher than the average 30% response rate to online surveys [9, 10]. The majority of respondents were from academic institutions, were located in a hospital and employed recent pediatric residency graduates. It is unclear if the respondents are overall representative of the larger surveyed population. In addition, in order to reach the most pediatric urgent care center directors possible, we administered the survey to the members of PEMNAC and SPUC, contributing to the limitation of targeting mainly academic urgent care centers and urgent care centers in urban and suburban areas. Another limitation of this study is that the respondents are all in senior leadership positions, which may introduce bias as leaders in urgent care may have self-interest in differentiating their field as a specialty. No recent pediatric residency graduates or trainees were included in this survey. It is possible that recent pediatric residency graduates and trainees would identify different degrees of competency and different knowledge gaps than their supervisors. In addition, in order to maintain anonymity, we did not ask respondents to specify the location of the UCC they direct. It is therefore possible that the majority of our results are from one region in the United States and not representative of pediatric UCCs around the country. Lastly, in an effort to keep the survey short and therefor maximize the likelihood of survey completion, we did not specify particular procedures or clinical competencies. It is possible the results would have varied from one procedure or clinical competency to the next, giving more granularity to the information.

## Conclusion

The majority of academic urban and suburban urgent care directors do not perceive recent pediatric residency graduates as ready to provide competent care in an UCC without further training. This training can likely be accomplished with 6 months to 1 year of additional training in clinical and procedural skills. Future research should be directed at determining specific subsets of skills needed and most effective training methods.

## Additional file

Additional file 1: Survey for urgent care directors. Includes the 10-question survey used for the needs-assessment study. (DOCX 17 kb)

## Data Availability

The datasets used and/or analysed during the current study are available from the corresponding author on reasonable request.

## Abbreviations

PEM: Pediatric Emergency Medicine
UCA: Urgent Care Association
UCC: Urgent Care Center
