# Supplementary material for: Pediatric urgent care education: a survey-based needs assessment
Source: BMC Health Serv Res. 2019 Jun 14;19:388. doi: 10.1186/s12913-019-4241-8 (PMC6570895; doi:10.1186/s12913-019-4241-8)
Supplement: Supplementary file 1 — Survey for urgent care directors. Includes the 10-question survey used for the needs-assessment study. (DOCX 17 kb) [file 12913_2019_4241_MOESM1_ESM.docx]

**Page 1: Site Characteristics**

Q1: How would you describe your site?

- Free-standing UCC not associated with academic institution
- Free-standing UCC associated with academic institution
- UCC located within hospital at an academic institution
- Other (please specify)

Q2: Please describe the training of your providers (select all that apply):

- Pediatrics residency
- Family Medicine residency
- Emergency Medicine residency
- Pediatric Emergency Medicine fellowship
- Other (please specify)

Q3: Please describe your location:

- Urban
- Suburban
- Rural
- Other (please specify)

**Page 2**

Q1: Did you advertise for or hire a general pediatrician in your ED or urgent care center in the past year?

- Yes
- No

Q2: How difficult was it to recruit a general pediatrician in your ED or urgent care center?

- Very difficult
- Somewhat difficult
- Somewhat easy
- Very easy

Q3: How comfortable do you think recent pediatric residency graduates are with the diagnosis and management of common ED/urgent care clinical cases?

- Very uncomfortable
- Somewhat uncomfortable
- Somewhat comfortable
- Very comfortable
- I haven’t worked with recent pediatric residency graduates

Q4: How comfortable do you think recent pediatric residency graduates are with common ED/urgent care procedures?

- Very uncomfortable
- Somewhat uncomfortable
- Somewhat comfortable
- Very comfortable
- I haven’t worked with recent pediatric residency graduates

**Page 3**

Q5: How many months does it take for a recent pediatric residency graduate to demonstrate competence in the diagnosis and management of common ED/urgent care clinical cases and in the performance of common ED/urgent care procedures?

[free text response]

Q6: How important do you consider additional training/experience in urgent care medicine when hiring a pediatric residency graduate to work in your ED/urgent care center?

- Very unimportant
- Unimportant
- Important
- Very important

**Page 4**

Please rank the following skill sets according to their importance in a pediatric urgent care provider (1= the most important skill)

Administration / operations / flow

Procedures (lacerations, foreign body removal, etc.)

Clinical competency

Teaching

Quality Improvement

The last question is about skills needed to be an Urgent Care Center director. List any other skills that you felt were important during your development as an Urgent Care Center director.

[free text response]
